# Effect of Bioaugmentation on Biogas Yields and Kinetics in Anaerobic Digestion of Sewage Sludge

**DOI:** 10.3390/ijerph15081717

**Published:** 2018-08-10

**Authors:** Magdalena Lebiocka, Agnieszka Montusiewicz, Agnieszka Cydzik-Kwiatkowska

**Affiliations:** 1 Faculty of Environmental Engineering, Lublin University of Technology, 40B Nadbystrzycka, 20-618 Lublin, Poland; a.montusiewicz@pollub.pl; 2 Faculty of Environmental Sciences, University of Warmia and Mazury in Olsztyn, 45g Słoneczna, 10-709 Olsztyn, Poland; agnieszka.cydzik@uwm.edu.pl

**Keywords:** anaerobic digestion, wastewater sludge, bioaugmentation, metagenome, NGS

## Abstract

Bioaugmentation with a mixture of microorganisms (Bacteria and Archaea) was applied to improve the anaerobic digestion of sewage sludge. The study was performed in reactors operating at a temperature of 35 °C in semi-flow mode. Three runs with different doses of bioaugmenting mixture were conducted. Bioaugmentation of sewage sludge improved fermentation and allowed satisfactory biogas/methane yields and a biodegradation efficiency of more than 46%, despite the decrease in hydraulic retention time (HRT) from 20 d to 16.7 d. Moreover, in terms of biogas production, the rate constant k increased from 0.071 h^−1^ to 0.087 h^−1^ as doses of the bioaugmenting mixture were increased, as compared to values of 0.066 h^−1^ and 0.069 h^−1^ obtained with sewage sludge alone. Next-generation sequencing revealed that *Cytophaga* sp. predominated among Bacteria in digesters and that the hydrogenotrophic methanogen *Methanoculleus* sp. was the most abundant genus among Archaea.

## 1. Introduction

The development of wastewater treatment technology, together with the implementation of environmental legislation, has successfully protected the aquatic environment from pollution. However, at the same time, sewage sludge is generated as the by-product of the wastewater treatment. Sewage sludge is becoming a worldwide environmental problem because of its increasing production and its high contents of organic matter, pathogens, and heavy metals. 

Anaerobic digestion is a biological process that can degrade organic material by the concerted action of a wide range of microorganisms in the absence of oxygen. However, the advantages of the anaerobic digestion process in the treatment of sewage are still far from being optimized. Regardless of the temperature conditions, only around 50% to 60% of the organic matter can be degraded, leaving a large potential of increasing the biogas production [1]. A better understanding of the basic mechanisms occurring in the digester, conducting the process at high temperatures, application of different kinds of pre-treatment methods (freezing/thawing; cavitation), phase separation, and, recently, bioaugmentation has been applied to improve the anaerobic digestion. 

Bioaugmentation is the addition (augmentation) of specialized microbial cultures which are typically grown separately under well-defined conditions to perform a specific task in a given environment (in situ or in a bioreactor) [2,3]. This approach has been used for hazardous waste remediation, as well as for the biodegradation of wastewater in wastewater treatment plants and many others biological treatment processes [4]. In aerobic wastewater treatment, bioaugmentation has resulted in more reliable nitrification, biological phosphorous removal, improved sludge settling, enhanced grease degradation, and accelerated transformation of xenobiotic organic contaminants, such as pentachlorophenol [5,6,7,8]. Bioaugmentation has also been studied at laboratory scale to increase the methane production during the anaerobic digestion of animal manure [9], cellulose-rich [10,11], and lipid-rich waste [12] as well as seed biomass [13]. Schmidt et al. [14] used bioaugmentation during the anaerobic digestion of sewage sludge to improve the polycyclic aromatic hydrocarbons removal. Moreover, the bioaugmentation of anaerobic digestion communities by the adapted hydrolytic consortia increased the biogas yield by 10–29% in the anaerobic digestion of maize silage [15]. 

Interestingly, bioaugmentation was investigated as a method of decreasing the recovery period of anaerobic digesters exposed to a transient toxic event [16,17]. The comprehensive review by Tale et al. [18] described the beneficial effects of bioaugmentation on the efficiency of biochemical processes under temporary organic overloading of reactors. Bioaugmentation has been considered as a useful strategy, playing an excellent role for performance enhancement and recovery in biosystems under various stresses due to the improvement of detrimental conditions, retention, and adaptation of bioaugmentation consortium. However, bioaugmentation faces the potential risk of functional failure, even negative effects on biosystems [19]. Failure of bioaugmentation has been reported to be associated with numerous factors that include the growth rate being lower than the rate of washout, insufficient inoculum size, and substrate availability. Limitations of bioaugmentation can be overcome through the techniques that include increased inoculum dosing, a longer period of inoculum acclimatization in reactors, the addition of nutrients and surfactants, and application of sufficient acclimatization periods. Surveys of the literature show that a key area for the further research should be towards acquiring a better understanding of the degradation pathways where bioaugmentation is applied [20,21,22]. The present study examines the influence of bioaugmentation on the efficiency of anaerobic digestion of sewage sludge from a municipal wastewater treatment plant. The novel aspect of the study involved using a mixture of wild-living Bacteria and Archaea from Yellowstone National Park, USA, for bioaugmentation. The effect of this bioaugmentative mixture on the kinetics of biogas production and the microbial structure in semi-flow anaerobic reactors was determined.

## 2. Materials and Methods

### 2.1. Material Characteristics

Sewage sludge that included two-source residues (originated from primary and secondary clarifiers) were obtained from the Puławy municipal wastewater treatment plant (WWTP), Poland. Sludge was sampled once a week in the WWTP. Under laboratory conditions, the sludge was mixed at the volume ratio of 60:40 (primary: waste sludge), then homogenized, manually screened through a 3 mm screen and partitioned. The sludge samples were stored at 4 °C in a laboratory fridge for no longer than one week. Sludge prepared in this manner was fed to the digester as mixed sewage sludge (SS). The main characteristics of SS during the entire cycle of experiments was as follows (the average value and standard deviation are given): chemical oxygen demand (COD)—43.00 ± 5.49 g dm^−3^, volatile fatty acids (VFA)—1179 ± 733 mg dm^−3^, total solids (TS)—37.0 ± 2.9 g kg^−1^, volatile solids (VS)—28.6 ± 2.11 g kg^−1^, pH—6.75 ± 0.33, and alkalinity—846 ± 268 mg CaCO_3_ dm^−3^.

**Figure 1:**
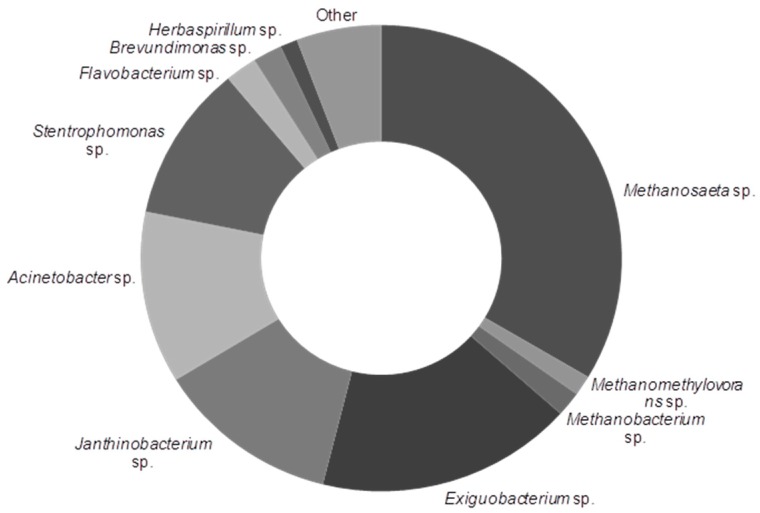
Microbial genera present in the bioaugmenting substrate; “other” includes unclassified bacteria (1%) and Archaea (1%), and taxa with abundance below 1% (3%); sequencing results are present in the Sequence Read Archive SRA (National Center for Biotechnology Information NCBI, BioProject PRJNA431048).

A liquid solution containing a mixture of Bacteria and Archaea was prepared in a continuous mode throughout the experiment. A nylon pouch filled with a powdery substrate (ArcheaSolutions Inc., Evansville, IN, USA) was placed inside a generator. The microbial composition of the powdery substrate after 1 day of cultivation at 37 °C under constant mixing conditions in distilled water showed that about 36% of microorganisms belonged to Archaea with *Methanosaeta* as s predominant genus and that about 59% of microorganisms belonged to Bacteria with *Exiguobacterium*, *Janthinobacterium*, *Acinetobacter,* and *Stenotrophomonas* as the most abundant genera (Figure 1). 

The analysis of the substrate was performed as described in *Molecular analysis*, sequences were placed in NCBI (BioProject PRJNA431048). The substrate was packed in a vinyl alcohol coating, which dissolved upon contact with dechlorinated pipe water flowing through the device. The release of an appropriate microbial content of 1.08 g L^−1^ h ^−1^ required a continuous flow of water at a level of about 0.5 L min^−1^. After 30 days, the pouch containing a mixture of microorganisms was replaced by a new one. The generator was linked to two serially-connected storage tanks with a total active volume of 320 L. The liquor prepared in this manner was stored at room temperature. The average total solids (TS) of the liquor differed slightly during the experiments and reached 0.48 and 0.45 g kg^−1^ in phase 1 and phase 2, respectively, while the average volatile solids (VS) oscillated around 0.04 g kg^−1^. The COD values were 22 g·m^−3^ in phase 1 and 26 g·m^−3^ in phase 2. Similarly, the VFA concentrations were 21 and 24 g·m^−3^, respectively. The alkalinity of 330 g CaCO_3_·m^−3^ and pH value of 7.16 were obtained for both phases.

### 2.2. Laboratory Installation and Operational Set-Up

**Figure 2:**
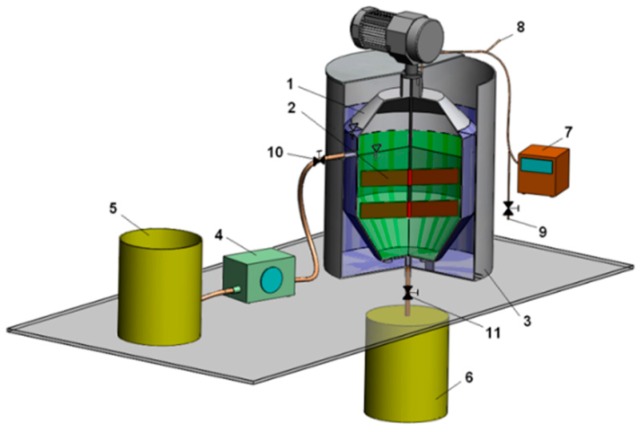
Laboratory installation. 1—anaerobic reactor, 2—mechanical stirrer, 3—heating jacket, 4—influent peristaltic pump, 5—influent storage vessel, 6—effluent storage vessel, 7—drum gas meter, 8—gaseous installation and gas sampler with a rubber septum, 9—dewatering valve, 10—inlet valve, 11—outlet valve.

The study was performed in anaerobic reactors operating at a temperature of 35 °C in semi-flow mode. The laboratory installation consisted of three completely mixed digesters (with an active volume of 40 dm^3^) working in parallel, equipped with a gaseous installation (consisting of pipelines, pressure equalization unit and a mass flow meter), an influent peristaltic pump and storage vessels (Figure 2).

An inoculum for the laboratory reactors was taken from Puławy wastewater treatment plant as a collected digest from a mesophilic anaerobic digester with a volume of 2500 m^3^ that was operated at 35–37 °C and a hydraulic retention time (HRT) of about 25 days. The adaptation of the digester biomass was achieved after 30 days. 

Two phases of the experiment were conducted, differing in the organic loading rates (OLRs). Each phase lasted 90 days (30 days for acclimatization and 60 days for measurements). In the first phase, three reactors were operated. The first reactor (R1—control one) was fed daily with 2 dm^3^ of mixed sludge. The HRT reached 20 days, and the OLR was time-dependent with an average value of 1.55 kg VS m^−3^ day^−1^.

The second reactor (R2) was operated following the same schedule. However, the reactor was fed with sludge bioaugmented with microbial mixture at a volumetric ratio of 91:9 (the influent consisted of a mixture of 2 dm^3^ sludge and 0.2 dm^3^ of bioaugmenting mixture). The HRT was shortened to 18.2 days, and the average value of the OLR was 1.54 kg VS m^−3^ day^−1^.

The third reactor (R3) arrangement was the same as in R2, but this time the volumetric ratio was set at 87:13 (influent consisted of a mixture of 2 dm^3^ sludge and 0.3 dm^3^ of bioaugmenting mixture). The HRT shortened to 17.4 days, and the average value of the OLR was 1.53 kg VS m^−3^ day^−1^.

In the second phase, two reactors were operated. The fourth reactor (R4) was operated analogously to the R1 (as a control), although at OLR of only 1.30 kg VS m^−3^ day^−1^.

Reactor R5 was operated also at low OLR of 1.33 kg VS m^−3^ day, and the sludge to bioaugmenting mixture volumetric ratio was set at 83:17 (influent consisted of a mixture of 2 dm^3^ sludge and 0.4 dm^3^ of bioaugmenting mixture). The HRT was shortened to 16.7 days.

The reactors were bioaugmented in continuous mode because of the long-term wide microbial growth (from 20 h to 20 days) as well as their possible wash out from the system.

### 2.3. Analytical Methods

In the mixed sludge (SS), total chemical oxygen demand (COD), total solids (TS) and volatile solids (VS), were analyzed once a week. The same schedule was used for determining the values of the parameters that characterized the supernatant (sludge liquid phase) before digestion. These parameters included soluble chemical oxygen demand (SCOD), volatile fatty acids (VFA), alkalinity and pH level. The supernatant samples were obtained by centrifuging the sludge at 4000 rpm for 30 min. All analyses were carried out in accordance with the procedures listed in the Standard Methods for the Examination of Water and Wastewater [23]. 

The bioaugmenting mixture was examined twice a week as an averaged and a collected sample taken from its storage tank. The following parameters were analyzed: COD, TS, VS, VFA, alkalinity, and pH.

In the digested sludge, the specified parameters were determined two times a week, in accordance with the timetable adopted. Similarly, the supernatant of the digested sludge was examined using the same schedule.

Anaerobic digestion efficiency was controlled by the daily evaluation of the biogas yield and its composition (CH_4_, CO_2_, and other gases). Moreover, the volatile solids removal efficiency was evaluated according to the American Public Health Association (APHA) [23].

The biogas production was determined using Aalborg (USA) digital mass flow meter. Its composition was measured using Trace GC-Ultra gas chromatograph coupled with a thermal conductivity detector (TCD) fitted with divinylbenzene (DVB) packed columns. The Rt-Q-Bond column was used to determine the CH_4_ and CO_2_ concentrations. The parameters used for the analysis were as follows—injector 50 °C and detector 100 °C. The carrier gas was helium with a flux rate of 1.5 cm^3^·min^−1^. Peak areas were determined by the computer integration program (CHROM-CARD).

The biogas production curves were made on the basis of the averaged experimental data acquired from an XFM Control Terminal. Both the rate constant k and maximum biogas production V_max_ were obtained by means of nonlinear regression method with Statsoft Statistica software (version 10, TIBCO Software Inc., Palo Alto, CA, USA). The strength of the relationships between groups of the results was determined using Pearson’s correlation coefficient (R) and determination coefficient R^2^.

### 2.4. Molecular Analysis

Metagenome of the digesters in the first phase was analyzed after 90 days of each reactor operation. The collected samples of biomass were stored at −20 °C. After thawing, DNA was isolated from the biomass using GenElute™ Bacterial Genomic DNA Kit (Sigma-Aldrich Chemie Gmbh, Munich, Germany) according to the producer’s protocol, including lysozyme digestion. The presence of DNA was confirmed by agarose electrophoresis. Purity and concentration of the isolated DNA were measured spectrophotometrically using a Biophotometer (Eppendorf, Hamburg, Germany). A universal 515F (GTGCCAGCMGCCGCGGTAA) and 806R (GGACTACHVGGGTWTCTAAT) primer set was used to amplify the archaeal and bacterial 16S rDNA gene. Next-generation sequencing (NGS) using the MiSeq Illumina platform was applied to sequence the amplicons. The sequencing was performed in the Research and Testing Laboratory (USA). Over 126 thousands of full sequences were obtained.

The sequences were analyzed bioinformatically as described in Świątczak et al. [24]. In short, to detect and remove chimeras from the raw reads, UCHIME [25] was applied. The readouts were condensed into FASTA format and clustered into operational taxonomic units (OTUs) using USEARCH global alignment [26]. A .NET algorithm utilizing BLASTN+ was used to query FASTA formatted file with seed sequences for each cluster against a database of NCBI derived sequences. Sequences were aligned by Infernal [27] and clustered by Complete Linkage Clustering (a module of the RDPipeline, https://rdp.cme.msu.edu/). The Shannon-Wiener index of diversity (H’) [28] was calculated (at a level of species). Samples were from the same run and have a similar number of reads; therefore, normalization was not performed to avoid data loss. Rarefaction analysis was performed using a module of the RDPipeline. The sequences have been deposited in the Sequence Read Archive (SRA) NCBI within BioProjectPRJNA380917 as an experiment ‘Metagenome of bioaugmented anaerobic digesters.’ 

## 3. Results and Discussion

### 3.1. Impact of Bioaugmentation on the Efficiency of Anaerobic Digestion of Sewage Sludge

**Figure 3:**
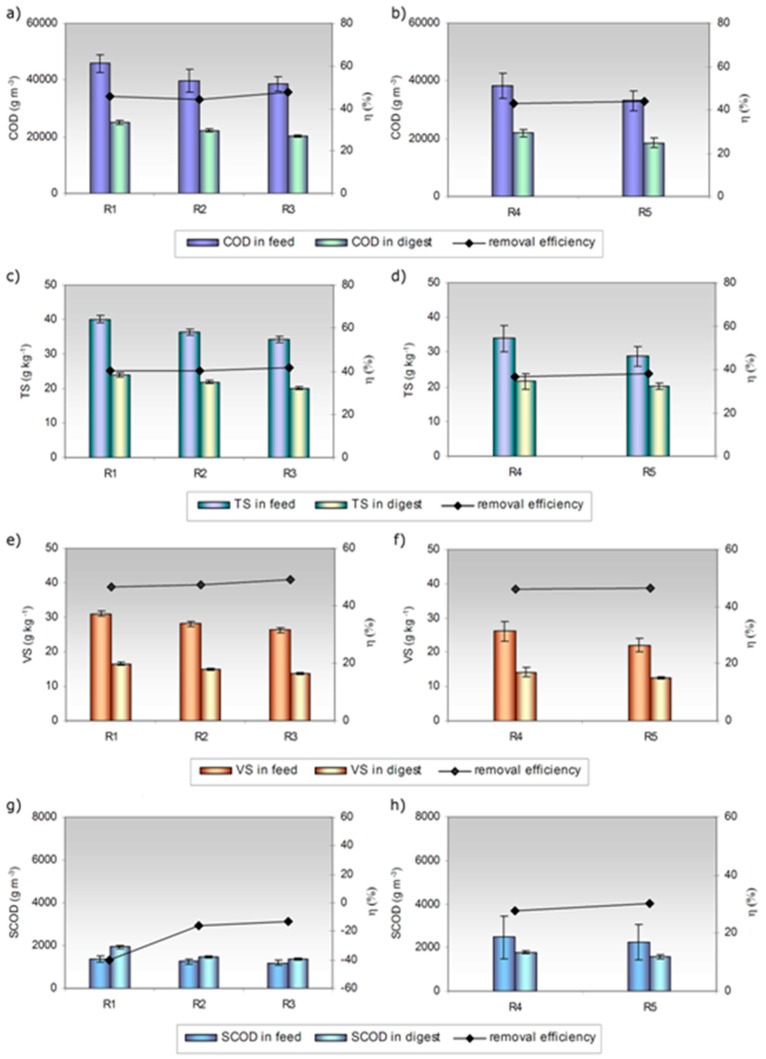
Concentration of organic compounds (expressed as chemical oxygen demand (COD), total solids (TS), volatile solids (VS), and soluble chemical oxygen demand (SCOD)) in feed and digest, as well as related removal efficiencies (average values are given, error bars represent 95% confidence limits for means): (**a**) COD changes in phase 1, (**b**) COD changes in phase 2, (**c**) TS changes in phase 1, (**d**) TS changes in phase 2, (**e**) VS changes in phase 1, (**f**) VS changes in phase 2, (**g**) SCOD changes in phase 1, (**h**) SCOD changes in phase 2.

The characteristics of both the mixtures feeding the reactors and the digests are presented in Figure 3. As indicated in the figure, the characteristics of SS varied in the control reactors (R1 and R4), which probably resulted from the changes in the chemical composition of sewage (Figure 1, Table 1).

Bioaugmentation by a mixture of Bacteria and Archaea decreased all parameters in the feedstock (due to dilution) as compared to sewage sludge. Decreases in COD concentration from 45.8 g dm^−3^ to 39.8 g dm^−3^ and 38.6 g dm^−3^, VS from 31.0 g kg^−1^ to 28.1 and 26.4 g kg^−1^, and TS from 40.1 g kg^−1^ to 36.4 g kg^−1^ and 34.2 g kg^−1^ were observed for R1, R2, and R3, respectively. The concentration of SCOD in the bioaugmented reactors was 1.25 and 1.20 g dm^−3^ and was lower than that in the control (1.40 g dm^−3^). Similarly, in R4 and R5, the concentration of VS and TS decreased by 15% (from 26.1 to 22.1 g kg^−1^ and from 33.9 to 28.8 g kg^−1^, respectively), COD by 13.5% (from 38.3 to 33.1 g dm^−3^), and SCOD by 9% (from 2.5 to 2.3 g dm^−3^). The higher the dose of the bioaugmenting mixture, the greater the decrease in the aforementioned characteristics as compared to the control reactors.

The degrees of removal pertaining to VS and other parameters were used to evaluate the process efficiency. On average, the removal efficiencies of VS in R2, R3, and R5 (the bioaugmented reactors) amounted to 47.3%, 49.2%, and 46.4%, respectively. These values were greater than the ones obtained for sewage sludge (Figure 3); however, the difference was not statistically significant. The highest removal of VS was observed in the presence of 13% *v*/*v* dose of the bioaugmenting mixture (R3), despite the shortening of HRT from 20 to 17.4 d. On the other hand, the removal efficiency of TS reached 40.5%, 41.7%, and 37.9% in R2, R3, and R5, respectively; for the control reactors, it was slightly lower, equaling 40.0% and 35.6% for R1 and R4 control reactors, respectively. As far as the removal efficiency of COD is concerned, comparable values were observed for all reactors. However, the maximum COD removal efficiency, reaching 47.6%, was obtained with 13% *v*/*v* dose of the bioaugmenting mixture (R3). This observation is in line with the findings of Yu et al. [29], who reported that the application of bioaugmentation during the mature landfill leachate treatment slightly improved the COD removal efficiency. Interestingly, visible differences were observed for the efficiency of SCOD removal (Figure 3g,h). In comparison with the non-bioaugmented reactors, the SCOD removal efficiency increased by 24% and 27%, in the reactors (R2, R3, R5) supplemented with 9% and 13% of bioaugmenting mixture. In contrast, in the presence of 17% *v*/*v* of bioaugmenting mixture, the increase was only by 2.5%. To sum up, the removal efficiency of organic compounds increased, although the hydraulic retention time was shortened from 20 to 16.7 d. Hailei et al. [30] also reported that the addition of mixed microorganisms could shorten the sludge acclimation time, as well as improve the treatment efficiency.

### 3.2. Process Stability

To evaluate the stability of anaerobic digestion, pH, alkalinity, concentration of VFA and the VFA/alkalinity ratio in digest supernatant should be analyzed. The average values of these parameters in the feedstock and digest are given in Table 1. 

The results indicate that the pH of feedstock was at a comparable level in the bioaugmented and non-bioaugmented reactors. Conversely, alkalinity decreased in the bioaugmented reactors (R2, R3, and R5) by 5.5%, 8.4%, and 16.7%, respectively, but the differences were not statistically significant. An increase in pH was observed in the digest. The values for all runs were in a range favorable for methanogens, i.e., from pH 7.59 to pH 7.99. Similarly, the digest alkalinity increased more than 4-fold. In the bioaugmented reactors, pH and alkalinity were lower compared with the sewage sludge, and these differences were statistically significant. Bioaugmentation caused a decrease in the VFA of the mixture fed to the reactor, but the difference was not statistically significant. Higher VFA removal was reported for anaerobic digestion of sewage sludge: 71.9%—in R1, and 90.2%—in R4. For bioaugmented reactors, these values were 68.8% (R2), 55.6% (R3), and 76.2% (R5). The digest was characterized by low concentrations of VFA—they did not exceed the value of 290 g m^−3^ in any reactor. In the presence of the bioaugmenting mixture, the VFA/alkalinity ratio increased to 0.071 and 0.087 (for 9% and 13 *v*/*v* dose, respectively) and 0.079 (17% *v*/*v*). For the sludge, the ratio equaled 0.063 in R1 and 0.041 in R4. The process was carried out under stable conditions in both phases.

### 3.3. Biogas Production

The results of the investigations are shown in Table 2 (average data are reported). It should be noted that the feed conditions varied through phases (R1–R3 and R4–R5), which was attributed both to the changes in sludge characteristics and the addition of microorganisms from the bioaugmenting mixture. In terms of organic matter removed via anaerobic digestion, regardless of the feedstock, the lowest biogas yield was observed for the removed COD, while the highest for the removed VS. Considering organic substances fed to the reactor, a higher biogas yield was noted in terms of VS and lower regarding TS (Table 2).

The addition of microorganisms from the bioaugmenting mixture had no significant influence on the biogas production (the differences of means were not statistically significant). However, the biogas yields, as well as daily biogas production, were enhanced compared to the control (particularly for R2 and R3). These occurred despite the progressive decrease in the hydraulic retention time (HRT) from 20 d to 16.7 d that led to the faster washing out of the microorganisms from the semi-flow system. Moreover, similar methane content was noted independently of the mixture dose. It was also shown that bioaugmentation enabled to obtain higher biodegradation efficiency regarding the VS removal within shorter HRT. The above suggests the beneficial bioaugmentation effect. The results achieved could be attributed to the increase in rates of both biogas production and organics decomposition. It was due to the differences between the microbial communities of the bioaugmented digesters and the enhanced activity of microorganisms involved in bioaugumentation of anaerobic digestion. This explanation is consistent with the research by Duran et al. [31] regarding bioaugmentation with selected strains belonging to *Baccillus* sp., *Pseudomonas* sp., and *Actinomycetes* sp. The slight increase in biogas yields throughout bioaugmentation could result both from the HRT shortening and the TS/VS feedstock dilution by bioaugmenting mixture. The second one followed by the procedure of mixture preparation recommended by ArcheaSolutions Inc. To significantly improve the biogas yields, the higher concentration of the bioaugmenting mixture would be profitable. This could be achieved in the future research using a microfiltration module. The study of Poszytek et al. [32] using a novel bacterial strain *Ochrobactrum* sp. POC9 for bioaugmentation of sewage sludge anaerobic digestion revealed much better results than the ones presented here. In that case, the cumulative biogas production increased by 22.06% compared to the control, although the study was conducted in batch mode.

### 3.4. Kinetics

**Figure 4:**
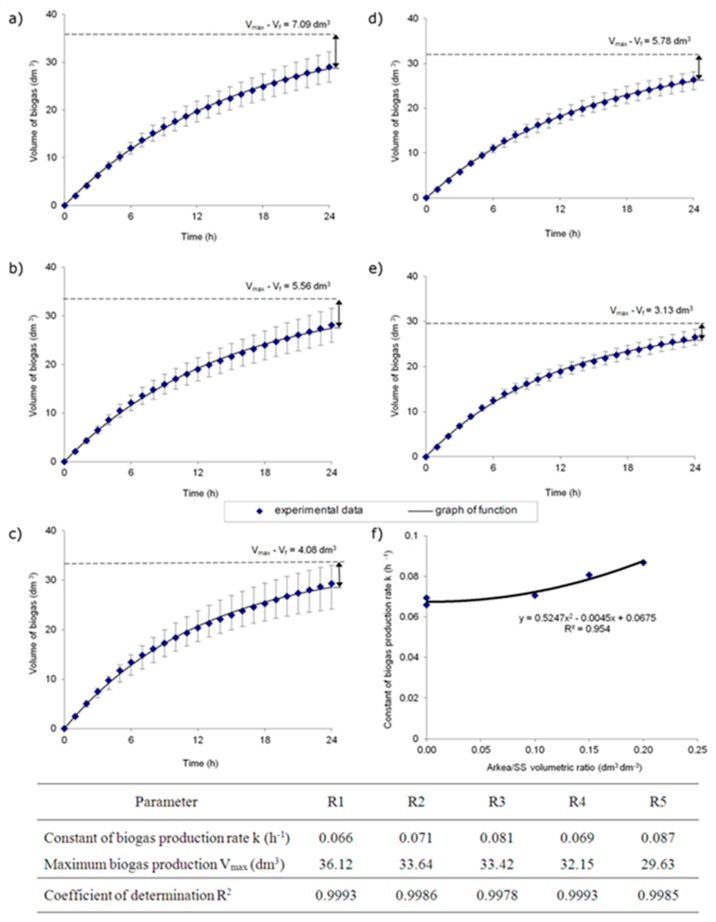
Biogas production in time (the average values from 30 measurement days and standard deviations are given), the values of kinetic constants and coefficients of determination for specified reactors: (**a**) R1, (**b**) R2, (**c**) R3, (**d**) R4, (**e**) R5, and (**f**) k constant as a function of Arkea/SS volumetric ratio.

In the quasi-flow system, the reactor was fed once a day with the portion of substrate or substrates and the same volume of digested medium was removed from the reactor. For this reason, the production of biogas between each feeding related to a temporal interval of 0–24 h. Accordingly, the reaction rate constant was expressed in hours, distinct from the typical units used for batch experiments (d^−1^). The average volume of biogas produced day by day (calculated for the exemplary 30 measurement days) is shown in Figure 4.

For a mathematical description of the changes in the biogas volume (V) produced in time (t), the most appropriate was the equation of first-order reaction [33] as V = V_max_ (1 − exp(−k·t)). This was confirmed by the determination coefficients (R^2^). The experimental data allowed to determine the reaction rate constant (k) and the maximum gas volume (V_max_), which theoretically can be derived from the feed portion feeding the reactor once a day.

The results indicate that the biogas production rate constants for sewage sludge were comparable and equaled 0.066 h^−1^ and 0.069 h^−1^ for R1 and R4, respectively. In the bioaugumented reactors, the k values were 0.071 h^−1^, 0.081 h^−1^, and 0.087 h^−1^ in R2, R3, and R5, respectively. It was probably caused by the enhanced microorganisms’ activity in the bioaugmented systems. This was confirmed by the increased biogas production rate constant k. The k value increased with the increasing the dose of bioaugmenting mixture, according to the equation y = 0.5247x^2^ − 0.0045x + 0.0675 (Figure 4f). 

In each phase, the differences between the maximum biogas production from the reactor feed (V_max_) and the actual value of biogas production obtained after the period of 24 h (V_f_) were determined (Figure 4). The difference V_max_ − V_f_ corresponds to the value of biogas potential in the digest and varied from 3.13 to 7.09 dm^3^ in the reactors. The best results were obtained in the bioaugmented reactors, with increasing doses of bioaugmenting mixture in the feedstock. In such cases, the untapped biogas potential decreased and amounted to 16.5% (R2), 12.0% (R3), and 10.6% (R5). In the control runs, the untapped biogas potential was higher and was up to 19.6% and 17.8% (R1 and R4, respectively). Bioaugmentation of digested sewage sludge resulted in an enhancement of the metabolism transformation rate, which was associated with the increase of process efficiency.

Importantly, the influence of the feed changes on the kinetics results determined on the basis of continuous data acquisition throughout experiment time was shown. This was revealed in terms of standard deviation growing within 24 h for the analyzed 30 measurement days.

### 3.5. Changes in Digest Morphology

The scanning electron microscopy was used to observe the microbial aggregates in the digest structure, the space relationship between microorganisms, as well as the presence of extracellular polymeric substances (EPS) and other materials.

**Figure 5:**
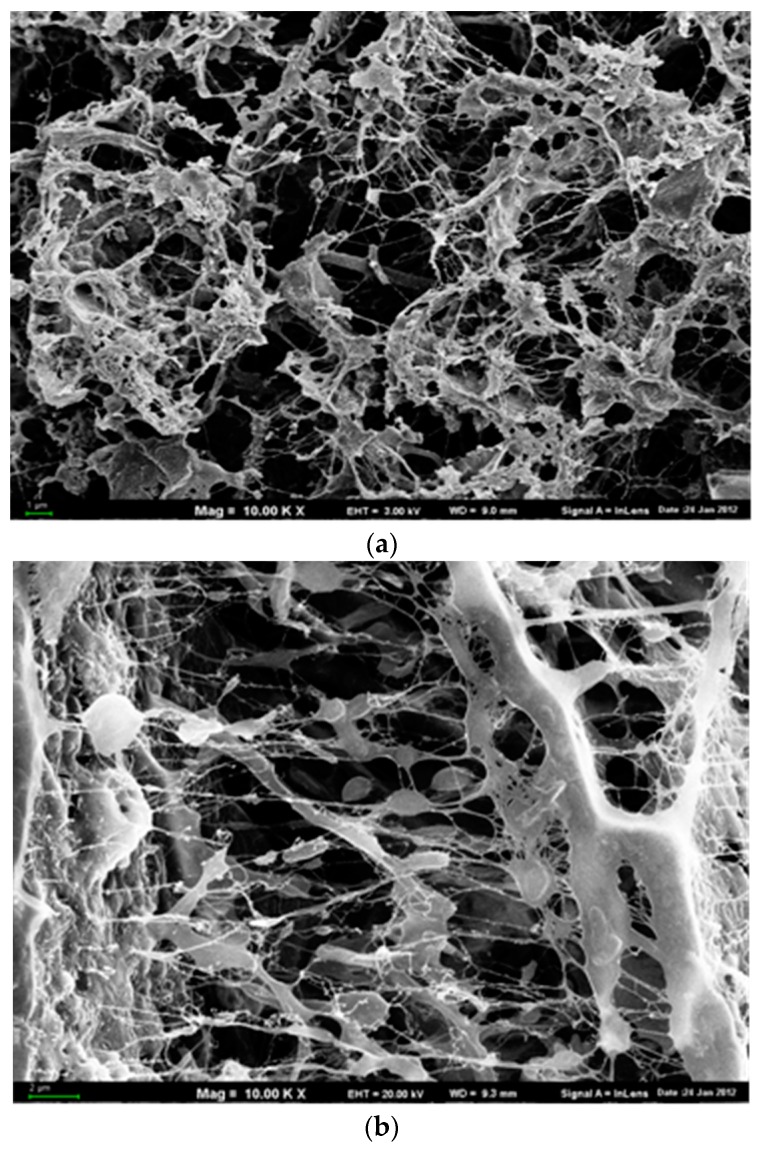
SEM micrograph of non-bioaugmented (**a**) and bioaugmented (**b**) digested sewage sludge (magnification 10×).

While analyzing the structure of the digested sludge, it was noted that despite the shortened HRT, larger agglomerates of microorganisms were formed in the bioaugmented reactors compared to the non-bioaugmented sludge (Figure 5). This was beneficial for the subsequent digest dewatering because of the extended sludge sediments capability (unpublished data). On the basis of the operation strategy ensuring comparable OLR value for both the bioaugmented and non-bioaugmented reactors, the observed effect could be largely attributed to extended EPS secretion by microorganisms [34,35] through bioaugmentation, and thus lower shear sensitivity and lower degree of dispersion [36]. According to the study of Yu et al. [37], the microbial community composition and its activity affected both the EPS production and composition. Interestingly, in the structure of the bioaugmented digest, the changes in the morphology of EPS were clearly visible. EPS began to collapse and condense into fiber-like structures [38]. Similarly, the differences referring to methanogens seem to indicate a response in their cell shapes to bioaugmentation that was reported by Zhang et al. [39].

### 3.6. Microbial Structure of Digesters

Sludge fermentation starts with hydrolysis followed by acidogenesis. In both these processes, the predominant microorganisms are obligatory anaerobes and facultative bacteria. Microorganisms conducting acetogenesis produce hydrogen and are in symbiosis with methanogenic archaea, which consume hydrogen (syntrophy or interspecies hydrogen transfer—IHT). The balance between methanogens and microorganisms involved in acido- and acetogenesis is crucial because if the activity of the latter is too high, anaerobic digestion will fail due to the acidification of the reactor. 

The microbial structure of the biomass from the reactors operated in the first phase of the experiment was analyzed using next-generation sequencing. Rarefaction analysis was used to characterize the richness of taxa in the experimental digesters. At the genus level, the curves leveled off, indicating acceptable sampling and coverage of the richness in the samples (data not shown). The microbial diversity of the biomass was the highest in the control reactor (H’ = 2.86) and decreased gradually to H’ = 2.52 and H’ = 2.16 in R2 and R3, respectively, as the dose of bioaugmenting mixture was increased. In the reactors, Bacteria predominated and Archaea constituted between 1.3% and 1.4% of identified sequences (Table 3). 

This value was one order of magnitude lower than for example the one noted in mesophilic full-scale digesters with sewage sludge [24]; however, it was similar to the values noted in mesophilic reactors for co-digesting of fish waste and cow manure (about 1%) [7] or solid-state digesters fed with kitchen waste, pig manure and excess sludge (about 0.5%) [40]. In this study, despite being only a small fraction of the entire microbial community, Archaea ensured efficient production of methane-rich biogas.

The structure of the biomass in the reactors differed from that of the mixture for bioaugmentation. This indicates that most of the microbes in the mixture were not able to survive in the reactors; however, the ones that survived improved the efficiency of methane fermentation as concluded based on the rate constant for biogas production and efficiency of SCOD removal. Within the biomass, the percentage of unclassified bacteria increased with increasing dose of bioaugmenting mixture showing that high diversity of yet unknown microorganisms was present in the reactor as a result of bioaugmentation.

Among Bacteria, the core communities belonged to the phyla Thermotogae, Spirochaetes, Cloacimonetes, Actinobacteria, Bacteroidetes, Chloroflexi, Firmicutes, and Proteobacteria. Bacteroidetes, Chloroflexi, Firmicutes, and Proteobacteria contain most of the identified species of acidogenic bacteria that support the hydrolysis stage [41]. Microorganisms belonging to class Cytophagia predominated in the biomass (12.3–18.9%). However, as doses of the bioaugmenting mixture were increased, the percentage of *Cytophaga* sp. decreased; this was a strong association, with an R^2^ value of 0.91, indicating that 91% of the variation in *Cytophaga* sp. abundance was associated with the dose of bioaugmenting mixture. Similarly, an increased dose of this mixture was associated with decreases in the percent abundance of the order Verrucomicrobiales (R^2^ = 1.00), the phyla Cloacimonetes (R^2^ = 0.83), and Choroflexi (R^2^ = 1.00), including order Anaerolineales (R^2^ = 0.99). Members of the genus *Cytophaga* are important for anaerobic decomposition of biopolymers, such as xylan or cellulose. *Cytophaga xylanolytica* is a mesophilic anaerobe that grows by fermentation of mono-, di-, and polysaccharides (but not cellulose) to acetate, propionate, succinate, CO_2_, and H_2_; xylan-grown cells of this species have xylanase and various glycosidase activities. *Cytophaga hutchinsonii* can rapidly digest crystalline cellulose without free cellulases or cellulosomes [42]. As extracellular polymeric substances may comprise up to 30% of activated sludge, these activities of *Cytophaga* sp. may be crucial for efficient degradation of organics in fermented sludge.

The abundance of *Pseudomonas* sp. increased from 0.4% in R1 to nearly 4% in bioaugmented R2 and R3 and it was the only identified genus whose abundance increased by such a large amount. Such an increase can be advantageous for anaerobic digestion because Duran et al. [31] observed that the presence of selected strains of genera *Pseudomonas, Bacillus,* and *Actinomycetes* improved the anaerobic digestion of biosolids, increasing net CH_4_ production by 29% and diminished odor formation. In addition, Xia et al. [43] reported that an increase in the proportion of some functional organisms, including *Pseudomonas* sp., led to an increase in the efficiency of anaerobic digestion when the proportion of more biodegradable, low molecular weight fractions (<20 kDa) was increased 10 times because of solubilization of some of the proteins, polysaccharides, nucleic acids, and humic-like substances. In our study, the concentration of low molecular weight SCOD in the digest was negatively correlated (R^2^ = 0.97) with the abundance of *Pseudomonas* sp. in the biomass in the bioaugmented reactors, indicating that this genus played an important role in SCOD degradation.

Syntrophic bacteria comprised a significant part of the biomass. From 3.6 to 7.1% of the identified sequences belonged to syntrophic prokaryotes from phylum Cloacimonetes. The analysis of the proteome of *Candidatus Cloacimonas acidaminovorans* indicated that this bacterium derives carbon and energy from the fermentation of amino acids and that it is a syntroph producing H_2_ and CO_2_ from formate [44]. On the other hand, the abundance of phylum Cloacimonetes was linked with lowered methane production in reactors fed with protein-rich substrates [7]. In this study, the abundance of other syntrophic microorganisms belonging to *Syntrophus* sp. and *Smithella* sp. was relatively stable in the reactors (1.4–1.8% and 0.7–0.9%, respectively). *Smithella* sp. are syntrophic acetogens involved in propionate degradation in anaerobic digesters, while *Syntrophus* sp. oxidates fatty acids and benzoate [41,45].

During the methane fermentation, Methanomicrobiales are usually less numerous than Methanosarcinales [8]. In this study, the predominance of Methanomicrobiales (about 1% of all identified sequences) in the species structure of Archaea indicated hydrogenotrophic methanogenesis as the main pathway of methane generation. Although *Methanosaeta* sp. predominated in the liquor used for bioaugmentation, *Methanoculleus* was the most abundant genus in the reactors. The idea of the study was to bioaugment the reactor with Archaea microorganisms to support methane production because this is the most critical step in the anaerobic digestion conducted by slow-growing microorganisms and prone to changes in environmental conditions. From the microbial analysis of biomass, it can be concluded that the biodiversity of bioaugmenting mixture reflecting the potential of different species to colonize the fermentation reactors was low (only 11 species with abundance over 0.5%). Despite this, bioaugmentation was successful in terms of the most abundant group that is *Methanosaeta* sp. belonging to Methanosarcinales that produce methane via an acetotrophic pathway. This genus was present in the experimental reactors, but its abundance was below 0.5%. *Methanosaeta* sp. is sensitive to OLR. They were abundant in the mesophilic fermentation reactors that were operated at an OLR of 1 kg COD m^−3^ day^−1^ but the increase in OLR to 2 kg COD m^−3^ day^−1^, at the maintained process temperature, caused their disappearance from the biomass [46]. In the present study, OLR was higher than the optimal for *Methanosaeta* sp.; therefore, although present in the biomass, they were not able to outcompete *Methanoculleus* sp. that predominated in the reactors. *Methanoculleus* sp. commonly occurs in digesters operated in meso- and thermophilic temperatures, including the digesters in which the process is supported by physico-chemical treatment, e.g., microwave radiation [46]. *Methanoculleus* sp. cope well with high OLRs, comprising over 40% of biomass during thermophilic co-digestion of manure and waste whey at an OLR of 60.4 g COD m^−3^ day^−1^ [40]. This fact may explain the predominance in the present study of this genera among Archaea in the experimental reactors operated at the higher OLRs. 

## 4. Conclusions

Bioaugmentation decreased the HRT from 20 d to 16.7 d, but despite this decrease, the observed daily biogas production, methane content in the biogas and the biogas yield per kg of VS were similar in bioaugmented and control reactors. With bioaugmentation, SCOD removal improved, especially in reactors operated at higher OLR, which can be attributed to the increase in the rate of biogas production. Regardless of loading, the value of k was higher in bioaugmented reactors than in control reactors. The structure of the biomass in all reactors was different from that of the mixture for bioaugmentation and bioaugmentation diminished species diversity. In all digesters, bacteria belonging to Thermotogae, Spirochaetes, Cloacimonetes, Bacteroidetes, and Proteobacteria predominated with cellulose-hydrolyzing *Cytophaga* as the most abundant genus. The abundance of *Pseudomonas* sp. increased as the dose of bioaugmentative mixture was increased. The predominance of *Methanoculleus* sp. among Archaea indicated that hydrogenotrophic methanogenesis was the main pathway of methane generation.

## Figures and Tables

**Table 1:** Volatile fatty acids (VFA) concentration, alkalinity and pH values in feed and digest for specified reactors.

Reactor	Value	VFA (g m^−3^)		Alkalinity (g CaCO_3_ m^−3^)		pH	
		Feed	Digest	Feed	Digest	Feed	Digest
R1	Average	838	235	812	3722	6.81	7.99
	lower/upper 95% mean	719/957	66/404	764/860	3614/3830	6.68/6.94	7.95/8.03
R2	Average	771	241	769	3391	6.83	7.86
	lower/upper 95% mean	678/864	112/370	732/806	3268/3514	6.70/6.96	7.80/7.92
R3	Average	660	291	749	3336	6.82	7.72
	lower/upper 95% mean	542/778	203/379	712/786	3229/3443	6.68/6.96	7.66/7.78
R4	Average	1520	149	880	3670	6.68	7.71
	lower/upper 95% mean	147/2893	115/183	610/1150	3524/3816	6.44/6.92	7.44/7.98
R5	Average	1062	253	754	3195	6.75	7.59
	lower/upper 95% mean	163/1961	167/339	578/930	3078/3312	6.53/6.97	7.37/7.81

**Table 2:** Biogas production and corresponding yields as well as methane content for bioaugmented and non-bioaugmented reactors.

Parameter	Unit	R1 (Control)	R2	R3	R4 (Control)	R5
Daily biogas production ^a^	dm^3^ d^−1^	23.23 ± 3.7 ^b^	24.26 ± 3.8	24.53 ± 3.8	19.59 ± 2.2	19.86 ± 2.1
Biogas yield	m^3^ kg^−1^ VS added	0.38 ± 0.07	0.40 ± 0.07	0.40 ± 0.07	0.38 ± 0.05	0.37 ± 0.05
	m^3^ kg^−1^ TS added	0.29 ± 0.05	0.31 ±0.05	0.31 ±0.05	0.29 ±0.04	0.29 ±0.04
	m^3^ kg^−1^ VS removed	0.83 ± 0.20	0.86 ± 0.21	0.84 ± 0.21	0.82 ± 0.22	0.83 ± 0.22
	m^3^ kg^−1^ TS removed	0.75 ± 0.18	0.78 ± 0.21	0.77 ± 0.19	0.79 ±0.27	0.78 ± 0.29
	m^3^ kg^−1^ COD removed	0.52 ± 0.09	0.53 ± 0.11	0.53 ±0.11	0.55 ± 0.09	0.55 ± 0.13
Methane content	%	56.25 ± 1.93	56.56 ± 1.58	56.16 ± 2.06	55.22 ± 1.98	55.57 ± 2.50

a—the average value, b—in normal conditions.

**Table 3:** Percentage of bacterial taxa in biomass from the experimental reactors.

Kingdom; Phylum; Class; Order; Family; Genus	R1	R2	R3
Bacteria; Verrucomicrobia; Verrucomicrobiae; Verrucomicrobiales; Unclassified	3.1	1.0	0.4
Bacteria; Unclassified	31.0	38.8	52.7
Bacteria; Thermotogae; Thermotogae; Thermotogales; Unclassified	4.4	2.8	4.2
Bacteria; Synergistetes; Synergistia; Synergistales; Synergistaceae; Synergistes	0.5	0.3	0.1
Bacteria; Spirochaetes; Unclassified	2.8	3.9	3.9
Bacteria; Spirochaetes; Spirochaetia; Unclassified	0.8	0.3	0.3
Bacteria; Proteobacteria; Unclassified	0.4	0.5	0.3
Bacteria; Proteobacteria; Gammaproteobacteria; Xanthomonadales; Xanthomonadaceae; *Thermomonas*	0.5	0.4	0.3
Bacteria; Proteobacteria; Gammaproteobacteria; Pseudomonadales; Pseudomonadaceae; *Pseudomonas*	0.8	3.9	3.6
Bacteria; Proteobacteria; Deltaproteobacteria; Syntrophobacterales; Unclassified; Unclassified	0.8	1.2	0.8
Bacteria; Proteobacteria; Deltaproteobacteria; Syntrophobacterales; Syntrophaceae; *Syntrophus*	1.4	1.8	1.7
Bacteria; Proteobacteria; Deltaproteobacteria; Syntrophobacterales; Syntrophaceae; *Smithella*	0.9	0.8	0.7
Bacteria; Proteobacteria; Deltaproteobacteria; Desulfobacterales; Desulfobacteraceae; *Desulfofaba*	0.7	1.3	1.0
Bacteria; Proteobacteria; Betaproteobacteria; Rhodocyclales; Rhodocyclaceae; *Dechloromonas*	0.5	0.4	0.3
Bacteria; Proteobacteria; Betaproteobacteria; Burkholderiales; Comamonadaceae; *Rhodoferax*	1.3	1.0	0.7
Bacteria; Proteobacteria; Betaproteobacteria; Burkholderiales; Comamonadaceae; *Diaphorobacter*	0.8	0.8	0.5
Bacteria; Proteobacteria; Betaproteobacteria; Burkholderiales; Comamonadaceae; *Acidovorax*	0.5	0.4	0.4
Bacteria; Firmicutes; Unclassified	0.8	0.7	0.3
Bacteria; Firmicutes; Clostridia; Clostridiales; Clostridiaceae; *Clostridium*	0.5	0.5	0.3
Bacteria; Cloacimonetes; Unclassified	7.1	6.5	3.6
Bacteria; Chloroflexi; Unclassified	1.2	0.7	0.5
Bacteria; Chloroflexi; Anaerolineae; Anaerolineales; Unclassified	1.4	0.8	0.4
Bacteria; Bacteroidetes; Sphingobacteriia; Sphingobacteriales; Unclassified; Unclassified	0.7	0.4	0.3
Bacteria; Bacteroidetes; Cytophagia; Cytophagales; Cytophagaceae; *Cytophaga*	18.9	16.8	12.3
Bacteria; Bacteroidetes; Bacteroidia; Bacteroidales; Bacteroidaceae; *Bacteroides*	1.9	0.7	0.6
Bacteria; Actinobacteria; Actinobacteria; Micrococcales; Intrasporangiaceae; *Tetrasphaera*	0.5	0.4	0.4
Bacteria; Actinobacteria; Actinobacteria; Micrococcales; Dermatophilaceae; *Dermatophilus*	1.1	0.8	0.7
Archaea; Unclassified	0.5	0.4	0.3
Archaea; Euryarchaeota; Methanomicrobia; Methanomicrobiales; Methanomicrobiaceae; *Methanoculleus*	0.8	1.0	1.0
Low abundance *	9.2	7.5	5.4
No Hit	4.5	3.5	2.1

* In the table only bacterial taxa with abundance over 0.5% were presented.
